# Amelioration of motor/sensory dysfunction and spasticity in a rat model of acute lumbar spinal cord injury by human neural stem cell transplantation

**DOI:** 10.1186/scrt209

**Published:** 2013-05-28

**Authors:** Sebastiaan van Gorp, Marjolein Leerink, Osamu Kakinohana, Oleksandr Platoshyn, Camila Santucci, Jan Galik, Elbert A Joosten, Marian Hruska-Plochan, Danielle Goldberg, Silvia Marsala, Karl Johe, Joseph D Ciacci, Martin Marsala

**Affiliations:** 1 Neuroregeneration Laboratory, Department of Anesthesiology, University of California, San Diego, 9500 Gilman Drive, 92093, La Jolla, CA, USA; 2 Department of Anesthesiology, School for Mental Health and Neuroscience, Maastricht University Medical Center, Universiteitssingel 40, 6229, ER Maastricht, The Netherlands; 3 Institute of Neurobiology, Slovak Academy of Sciences, Soltesovej 9, 04001, Kosice, Slovakia; 4 Institute of Biology and Ecology, Faculty of Science, Pavol Jozef Safarik University, Srobarova 2, 04154, Košice, Slovakia; 5 Neuralstem, Inc, 9700 Great Seneca Hwy, Rockville, MD 20850, USA; 6 Institute of Animal Physiology and Genetics, Czech Academy of Sciences, Rumburska 89, 277 21, Libechov, Czech Republic; 7 Department of Cell Biology, Faculty of Science, Charles University in Prague, Vinicna 7, 128 00, Prague, Czech Republic; 8 UCSD Division of Neurosurgery, University of California, San Diego, 9500 Gilman Drive, 92093, La Jolla, CA, USA; 9 Sanford Consortium for Regenerative Medicine, 2880 Torrey Pines Scenic Drive, 92037, La Jolla, CA, USA

**Keywords:** Spinal cord injury, Human neural stem cells, Spinal grafting, Functional recovery, Rat

## Abstract

**Introduction:**

Intraspinal grafting of human neural stem cells represents a promising approach to promote recovery of function after spinal trauma. Such a treatment may serve to: I) provide trophic support to improve survival of host neurons; II) improve the structural integrity of the spinal parenchyma by reducing syringomyelia and scarring in trauma-injured regions; and III) provide neuronal populations to potentially form relays with host axons, segmental interneurons, and/or α-motoneurons. Here we characterized the effect of intraspinal grafting of clinical grade human fetal spinal cord-derived neural stem cells (HSSC) on the recovery of neurological function in a rat model of acute lumbar (L3) compression injury.

**Methods:**

Three-month-old female Sprague–Dawley rats received L3 spinal compression injury. Three days post-injury, animals were randomized and received intraspinal injections of either HSSC, media-only, or no injections. All animals were immunosuppressed with tacrolimus, mycophenolate mofetil, and methylprednisolone acetate from the day of cell grafting and survived for eight weeks. Motor and sensory dysfunction were periodically assessed using open field locomotion scoring, thermal/tactile pain/escape thresholds and myogenic motor evoked potentials. The presence of spasticity was measured by gastrocnemius muscle resistance and electromyography response during computer-controlled ankle rotation. At the end-point, gait (CatWalk), ladder climbing, and single frame analyses were also assessed. Syrinx size, spinal cord dimensions, and extent of scarring were measured by magnetic resonance imaging. Differentiation and integration of grafted cells in the host tissue were validated with immunofluorescence staining using human-specific antibodies.

**Results:**

Intraspinal grafting of HSSC led to a progressive and significant improvement in lower extremity paw placement, amelioration of spasticity, and normalization in thermal and tactile pain/escape thresholds at eight weeks post-grafting. No significant differences were detected in other CatWalk parameters, motor evoked potentials, open field locomotor (Basso, Beattie, and Bresnahan locomotion score (BBB)) score or ladder climbing test. Magnetic resonance imaging volume reconstruction and immunofluorescence analysis of grafted cell survival showed near complete injury-cavity-filling by grafted cells and development of putative GABA-ergic synapses between grafted and host neurons.

**Conclusions:**

Peri-acute intraspinal grafting of HSSC can represent an effective therapy which ameliorates motor and sensory deficits after traumatic spinal cord injury.

## Introduction

Extensive experimental and clinical data show that the mechanisms leading to a clinically-defined loss of neurological function after spinal trauma can in general be considered in two categories. First is the pathology and corresponding functional loss resulting from a direct mechanical injury of axons at the injury epicenter, and second is a progressive appearance of secondary changes (local edema, hematoma, excitotoxicity and ischemia) which can evolve over hours to weeks after the initial impact (for review see Hagg and Oudega [1]). Consistent with our current knowledge of the mechanism which leads to the development of secondary post-injury cascade, the current experimental and clinical treatment strategies primarily focus on: I) improvement of local metabolism and blood flow (for example, through decompression therapy and hypothermia) [2,3]; and II) modulation of local inflammatory response (for example, with methylprednisolone) [4-7]. A separate group of experimental treatment modalities is aimed at improving the local neurotrophic activity at and around the injury epicenter with the primary goal of increasing the survival of partially injured axons and/or neurons. In this category of experimentation, besides the use of locally delivered trophic factors (such as brain-derived neurotrophic factor (BDNF)-, glial cell line-derived neurotrophic factor (GDNF)-, and fibroblast growth factor (FGF)-peptides or growth factors-gene-encoding vectors) [8], regionally grafted fetal or embryonic stem cell-derived neuronal precursors are frequently used [9-18].

Recently, well-defined protocols were developed which permit the isolation and long-term stable expansion of (non-immortalized) human fetal brain or spinal cord tissue-derived neural stem cells [19-24]. Using these protocols, continuing neurogenic potential, as evidenced by neuronal differentiation and the ability of differentiated neurons to generate action potentials *in vitro*, was documented at even high (>20) passage numbers [24,25]. Some of these lines were successfully used for: I) generation of good manufacturing practice (GMP)-grade clonally-derived cell lines; II) extensive pre-clinical evaluation using a variety of neurodegenerative small and large animal models; and III) subsequently used successfully in Phase I human clinical trials [26-28].

In our previous studies, we have extensively characterized the *in vivo* treatment effect after spinal grafting of (clinical) GMP (cGMP)-grade human fetal spinal cord-derived stem cells (NSI-566RSCs line) using a spinal ischemia model in rats and transgenic rat model of amyotrophic lateral sclerosis (ALS) (SOD1^G93A^). In those studies, we have shown that: I) grafting of NSI-566RSCs into lumbar spinal cord of adult Sprague–Dawley (SD) rats with previous spinal ischemic injury is associated with a progressive improvement of ambulatory function which correlates with long-term grafted cell survival and extensive neuronal differentiation [29]; and II) bilateral lumbar grafting of NSI-566RSCs in pre-symptomatic SOD1^G93A^ rats provides a transient functional benefit and suppression of α-motoneuron degeneration, that is, a protective effect which was absent in media-injected animals [30]. Using the same cell line, we have also demonstrated the optimal dosing regimen and safety after grafting into the lumbar spinal cord of immunosuppressed minipigs [29]. The dosing design defined in this pre-clinical minipig study was then subsequently used in a recently completed Phase I human clinical trial in ALS patients receiving lumbar and cervical grafts of NSI-566RSCs [27,31]. In a more recent study using an immunodeficient rat model of complete spinal cord Th3 transection, it was shown that NSI-566RSCs or rat embryonic neural precursor cells, embedded in a fibrin matrix with trophic factors and grafted one week after injury, were able to form functional relays. The formation of functional relays was validated behaviorally (BBB locomotor score), electrophysiologically (spinal cord evoked potentials), and histologically (host on graft and graft on host synapses) [32].

The goal of our present study was to characterize the effect of NSI-566RSCs grafted spinally in a clinically relevant L3 spinal compression model in continuously immunosuppressed adult SD rats. The presence of a treatment effect was assessed by analysis of I) motor and sensory function, II) myogenic motor evoked potentials (MEPs), III) spasticity response during computer-controlled ankle rotation, and IV) qualitative analysis of grafted cell survival and maturation.

## Methods

### Animals and surgeries

**Figure 1:**
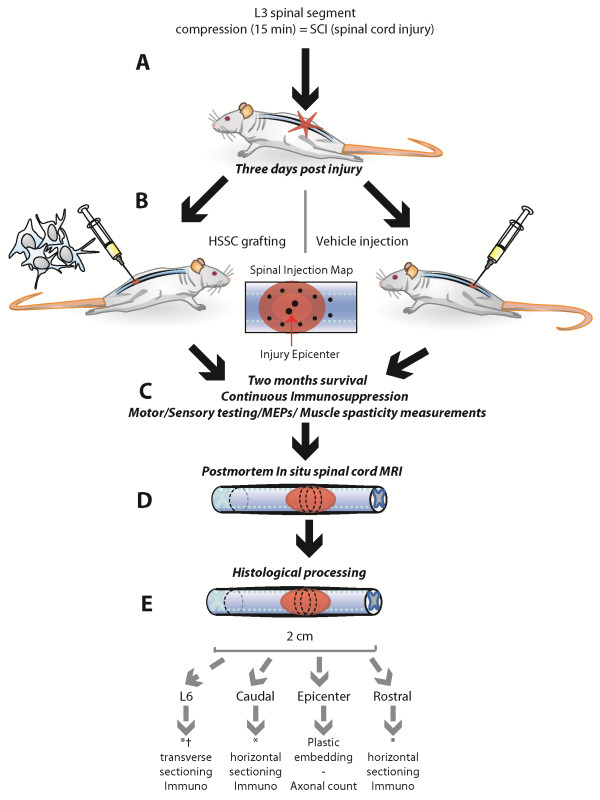
**Schematic diagram of experimental design. A:** To induce spinal cord injury, a 35 g circular rod was placed on the exposed L3 spinal segment and the spinal cord compressed in the dorso-ventral direction for 15 minutes. **B:** Three days after injury, the animals were randomly assigned to experimental groups and received a spinal graft of HSSC or media only. A total of 12 injections were performed targeting the injury epicenter and adjacent areas (see Spinal Injection Map). **C:** After spinal injections, the animals survived for two months while being continuously immunosuppressed and periodically tested for recovery of motor/sensory functions, changes in motor evoked potentials (MEPs) and gastrocnemius muscle spasticity response evoked by computer-controlled ankle rotation. **D:** At two months after treatment, animals were perfusion fixed with 4% PFA and spinal cord MRI-imaged *in situ* before histological processing. **E:** After MRI imaging, spinal cords were dissected from the spinal column and spinal blocks prepared for plastic embedding (injury epicenter region) or cryostat sectioning and used for immunofluorescence staining (the regions just above and below the injury epicenter). HSSC, human fetal spinal cord-derived neural stem cells; MRI, magnetic resonance imaging; PFA, paraformaldehyde.

All animal studies were approved by the University of California, San Diego Institutional Animal Care and Use Committee (Protocol No.: S01193). The study design is outlined in Figure 1. Twelve-week-old Female SD rats were used. The rationale for choosing female rats was based on our previous experience which demonstrates better tolerability of female rats to spinal trauma-related side effects, such as urinary retention. Animals were anesthetized with isoflurane (5% induction, 1.5% to 2% maintenance; in room air) and placed into a Lab Standard Stereotaxic frame (Stoelting, Cat# 51600, Wood Dale, IL, USA). The animal was elevated 2 cm by placing it on a homeothermic heating blanket (set at 37°C with feedback from a rectal thermometer (Harvard Apparatus, Cat# 507214, Holliston, MA, USA) which sits on a plastic rectangular block. The animal was then placed in Spine Adaptors (Stoelting, Cat# 51695, Wood Dale, IL, USA) and a wide Th13 laminectomy was performed using an air-powered dental drill and binocular microscope (exposing the dorsal surface of spinal segment L3). An acrylic rod (Ø 2.9 mm, length 15 cm; 35 g) was then slowly lowered onto the exposed L3 segment until it slightly touched the spinal cord but without inducing any compression. The laminectomy site was then filled with mineral oil in which the tip of a small thermocouple (Physitemp, Cat# IT-14, Clifton, NJ, USA) was submerged and touched the dura. The light from the two fiber optic light pipes of the surgical light (Fiber-Lite, Cat# MI-150 & BGG1823M, Dolan-Jenner, Boxborough, MA, USA) was focused on the surgical site (and directly illuminating the temperature probe). Next, the light intensity was manually regulated so that the spinal cord/mineral oil was warmed to 37°C and remained at 37 ± 0.3°C. If necessary, a 100 W infrared lamp was used to gradually adjust and maintain the animal’s core temperature at 37°C (rectal). When both temperatures (that is, paraspinal and rectal) were at 37 ± 0.3°C for at least five minutes, the rod was slowly lowered until its weight fully rested, perpendicularly, on the spinal cord. The rod was kept in place for 15 minutes, while both temperatures were maintained at 37 ± 0.3°C. After spinal compression, the rod and mineral oil were removed and the wound sutured in anatomical layers.

### Post-surgical care

Buprenorphine (0.05 mg/kg, s.c., Reckitt Benckiser, Richmond, VA, USA), 5 mL of lactated Ringer’s, 10 mg/kg of cefazolin (Novaplus/Sandoz, Holzkirchen, Germany) and standard triple antibiotic ointment to cover the incision site (bacitracin, neomycin, Polymyxin B) was given after every surgery. Bladders were manually emptied twice daily (if full). Sulfamethoxazole and trimethoprim USP oral suspension (200 mg and 40 mg per 250 mL drinking water, Hi-Tech Pharmacal, Amityville, NY, USA) was given for at least 10 to 14 days after spinal cord injury (SCI) or until autonomic bladder voiding occurred and for 1 to 2 days after any other surgery (sham or grafting). Food was provided by placing it at the bottom of cage and water bottles with an elongated drinking tube were used, until regular overhead supplies could be reached by the animal. Animals diagnosed with bacterial infections throughout the study were treated with sulfamethoxazole (as above), 10 mg/kg/day of cefazolin, and lactated Ringer’s 5 mL/0.5 day.

### Cell derivation and preparation

The cells, named ‘NSI-566RSC’, were produced by Neuralstem Inc. (Rockville, MD, USA), as described before [33]. Briefly, human spinal cord neural precursors (HSSC) were prepared from the cervical-upper thoracic region obtained from a single eight week fetus. The fetal tissue was donated by the mother in a manner fully compliant with the guidelines of NIH and FDA and approved by an outside independent review board and by the University of California, San Diego Human Research Protection Program (Project# 101323ZX). Meninges and dorsal root ganglia were removed and dissociated into a single cell suspension by mechanical trituration in serum-free, modified N2 media (human plasma apo-transferrin, recombinant human insulin, glucose, progesterone, putrescine, and sodium selenite in (Dulbecco’s) modified Eagle’s medium ((D)MEM)/F12). For growth of the HSSC, 10 ng/ml basic fibroblast growth factor (bFGF) was added to the modified N2 media and expanded serially as a monolayer culture on poly-D-lysine and fibronectin [34]. Approximately 6.1 × 10^6^ total cells were obtained upon the initial dissociation of the spinal cord tissue. The growth medium was changed every other day. The first passage was conducted 16 days after plating. At this point, the culture was composed mostly of post-mitotic neurons and mitotic HSSC. Mainly the mitotic cells were harvested through brief treatment with trypsin and subsequent use of soybean trypsin inhibitor. The cells were harvested at approximately 75% confluence, which occurred every five to six days (20 passages). At various passages, the cells were frozen in the growth medium plus 10% dimethyl sulfoxide at 5 to10 × 10^6^ cells/ml. The frozen cells were stored in liquid nitrogen. Upon thawing, the overall viability and recovery was typically 80% to 95%. A cell bank of passage 16 cells was prepared and used for this study.

For the production of eGFP-labeled NSI-566RSC, a Lentiviral vector was constructed containing the human Ubiquitin C promoter driving expression of enhanced GFP. Viral particles produced by infected 293FT cells were collected after overnight incubation, then concentrated by centrifugation and stored frozen. Neural stem cell cultures were infected by overnight incubation in growth medium supplemented with viral supernatant. Infected stem cells were washed with phosphate-buffered saline (PBS) and cultured as described above. After multiple passages, >90% of the cells were GFP positive (assessed after immunohistochemical staining). A cell bank of passage 17 cells was prepared and used for this study.

One day prior to each grafting day, one cryopreserved vial of the previously prepared cells was thawed, washed, concentrated in hibernation buffer, and shipped from the cell preparation site (Neuralstem, Inc., Rockville, MD, USA) to the surgery site (University of California, San Diego, CA, USA) at 2 to 8°C by overnight delivery. Upon receipt the following day, the cells were used directly for implantation without further manipulation. Before and after implantation, the viability of cells was measured with trypan blue (0.4%; Sigma-Aldrich, St. Louis, MO, USA). Typically, a >85% viability rate was recorded.

### Inclusion and exclusion criteria, randomization and blinding

Three days following SCI and prior to grafting, animals were randomly divided into three groups: the vehicle-injected group, non-injected group, or the HSSC-injected group. SCI animals with an open-field locomotion score of ≤1 and appearing healthy enough were included. Animals found moribund or automutilating at any point during the study were excluded and euthanized. A total of 42 animals were employed and divided into 6 experimental groups, as follows:

Group A (n = 14): SCI animals-NSI-566RSC-grafted,

Group B (n = 10): SCI animals-vehicle-injected,

Group C (n = 8): SCI animals-non-injected,

Group D (n = 6): sham operated (laminectomy only),

Group E (n = 6): naïve animals (no surgical manipulation)

Group F (n = 2): SCI athymic animals-ubiquitin.eGFP^+^ NSI-566RSCs-grafted.

One animal was excluded in Group A because of automutilation of the hind paw; two animals were excluded in Group C, one because of automutilation of the hind paw and one because of bacterial infection. Six animals had been replaced before dosing/randomization, five due to inadequate injuries and one because of bacterial infection.

### Grafting procedure

For the intraparenchymal injections, the animals were placed in the stereotactic frame as described above. The L3 spinal cord (that is, the dura mater) was then re-exposed at the previous laminectomy site. Injections were performed using a 33 gauge beveled needle and 100 μL Nanofil syringe (World Precision Instruments, Cat# NF33BV and Nanofil-100, Sarasota, FL, USA) connected to a microinjection unit (Kopf Instruments, Cat# 5000 and 5001, Tujunga, CA, USA). The duration of each injection was ≥45 seconds followed by a ≥30 second pause before slow needle withdrawal. The center of the injection was targeted intermediate of the ventral and dorsal horn and close to the lateral funiculus (distance from the dorsal surface of the spinal cord at the L3 level: 0.80 mm). Twelve injections (20,000 cells/μL) were done; four injections (0.5 μL each, 0.8 to 1.0 mm apart, rostrocaudally) at each lateral boundary of the injury (eight in total), plus two (bilateral) injections (0.5 μL each) 1.5 mm caudal from the previous, most caudal injections, and two injections at the core of the epicenter (1 μL at each side of the dorsal vein, bilaterally; see diagram in Figure 1). After the injections, the incision was cleaned with penicillin-streptomycin solution and sutured in two layers.

### Immunosuppression

Two days after injury (that is, one day before grafting), a methylprednisolone acetate (Depo-Medrol, 10 mg/kg, i.m.) was given, which was repeated thereafter three times with 1 mg/kg/week i.m. Starting directly after grafting, all animals received 1.5 mg/kg/BID s.c. of tacrolimus (Prograf/FK506, Astellas, Deerfield, IL, USA) until the end of the study. For post-transplant days 0 to 10, the animals also received 30 mg/kg/day s.c. of mycophenolate mofetil (CellCept, Genentech, CA, USA). Immunosuppression was also given to the non-grafted Sprague–Dawley animals (that is, the naïve, sham operated, and all SCI-control animals).

### Open field locomotion testing

Locomotion recovery after spinal cord contusion injury was monitored using a modified BBB open field locomotion rating scale [35]. The BBB score was modified to reflect the distinct locomotor recovery stages observed after L3 SCI. The modified score entailed eight well-defined degrees of locomotor recovery: 0 to 1: are identical to the BBB-score, 2: is cumulative score of 2 and 3 of the BBB score, 3: is cumulative score of 4, 5 and 6 of the BBB score, 4: is cumulative score of 7 and 8 of the BBB score, 5: reflects weight support with poor paw clearance, 6: is broadened and/or shortened stepping, and 7: is normal walking. In the present study, the locomotor score was obtained before grafting and weekly after injury until the end of the study (that is, 8.5 weeks post-injury). In addition to a modified BBB score, a regular full 21 scale BBB score was periodically assessed.

### Gait analysis

The CatWalk apparatus (CatWalk 7.1, Noldus Technology, Wageningen, The Netherlands) was used to quantify gait parameters during walkway crossings (for example, paw positioning, base of support, stride length, front limb versus hind limb coordination) by footprint analysis [36]. Animals had to walk down a horizontal glass walkway (109 × 15 × 0.6 cm, L × W × H), the glass of which is illuminated along the long edge. At the end of the walkway, animals had access to their home cage and were given a treat upon arrival (Certified Supreme Mini-Treats™, Cat# F05472-1, Frenchtown, NJ, USA). The light only enters the (side of the) glass and reflects merely internally (when the glass is bordered by air). As an animal walks on the glass walkway, light reflects off of the animal’s paws, producing a series of bright footprints when viewed through the glass, from below the walkway. The illuminated footprints were then recorded by a video camera with a wide-angle objective that was located underneath the elevated glass walkway. In order to get an optimal contrast between the paws and the surroundings; the test was performed in a room that was totally darkened. The animals were trained for smooth walkway crossing on the five days prior to the video acquisitions. To obtain accurate and meaningful data, the following criteria concerning walkway crossings needed to be met: (1) the animal needed to walk uninterrupted across the walkway, at a constant pace and (2) a minimum of three such crossings per animal were required. Animals without bilateral paw clearance could not be analyzed (n = 4 control-SCI animals, and 3 HSSC-treated animals). Digital data analysis consisted of assigning labels (left-fore, left-hind, right-fore, or right-hind) to the animal’s paw prints in a recorded walkway crossing, using dedicated CatWalk software. Next, the software calculated gait parameters. Data from three proper crossings were averaged for statistical analysis.

### Inclined ladder test

The inclined ladder test was performed as described before [37,38]. An inclined ladder (55°) with twenty 120 mm wide rungs (diameter: 1/4″), spaced at equal intervals (60 mm) and having 150 mm-high side walls was used. The rats were trained for this test so that smooth runs were recorded. At the end of the ladder, the animals had access to their home cage and received a treat (as above). The rats were placed at the bottom, and in front, of the ladder. The bottom of the ladder was placed on a 20 cm elevated platform. Climbing was video recorded from a position below the ladder, so that the ventral aspect of the animal is recorded. All animals were able to climb up the ladder. The correct placing of a hind paw and sustained position until its next forward move was counted over 18 rungs (placement on first and last rung were not counted).

### Single frame hind limb motion analysis

Two parameters were measured in bilateral video captures of animals crossing a runway: the foot-stepping angle (FSA) and the rump-height index (RHI), as described before [37,38]. The FSA is the angle at which the hind paw is placed on the ground just after the swing phase. The angle is defined by a line parallel to the dorsal surface of the paw and a horizontal line behind the paw. Four to six measurements were made for each hind limb (a total of 8 to 12 step cycles). The RHI was defined as the highest point of the base of the tail during the (recorded part of the) run. The values for the left and right paw of each animal were averaged. The elevated runway bar was made of a wooden plate/beam (1500 × 150 × 20 mm, L × W × H). The animals were trained to smoothly walk the beam. Once more, at the end of the beam the animals had access to their home cage and received a treat (as above). The videos (that is, the selected frames) were selected and analyzed using the video tool VirtualDub 1.9.11 (Written by Avery Lee, http://www.virtualdub.org) and the on-screen measurement tool Screen Ruler V1.0.1a (http://www.caveworks.net).

### Myogenic motor evoked potentials

Animals were anesthetized with ketamine (80 mg/kg i.p., Ketaset, Fort Dodge Animal Health, Overland Park, KS, USA). Myogenic motor evoked potentials (MEPs) were elicited by transcranial electrical stimulation (with a pulse duration of 1 ms at 7 mA using a DS3 constant current isolated stimulator (Digitimer LTD., Welwyn Garden City, UK) of the motor cortex using two percutaneously placed 30G stainless steel stimulation electrodes. Responses were recorded from the gastrocnemius muscle using 30G platinum transcutaneous needle electrodes (distance between recording electrodes approximately 1 cm; Grass Technologies, Astro-Med, Inc., West Warwick, RI, USA). Recording electrodes were connected to an active headstage (3110 W Headstage, Warner Instruments LLS, Hamden, CT, USA) and signal amplified using a DP-311 differential amplifier (Warner Instruments LLS). An amplified signal was acquired by the PowerLab 8/30 data acquisition system (AD Instruments, Inc., Colorado Springs, CO, USA) at a sampling frequency of 20 kHz, digitized and stored in a PC for analysis. MEPs were measured until the three to five highest (stable) recorded potentials were similar. Those traces were averaged per animal and multiplied by one thousand (μV; all values >1). Next, for data normalization, a logarithmical transformation was applied for further analysis (amplitudes of MEP traces tended to vary much more in animals with higher MEPs amplitudes).

### Measurement of muscle spasticity

At 1.5 weeks and 2 months post-injury, the presence of muscle spasticity in the lower extremities was measured using a previously described system [39]. Briefly, fully awake animals were placed in a restrainer and a hindpaw was taped to a rotational metal plate driven by a computer-controlled stepping motor. The metal plate is interconnected loosely to the ‘bridging’ digital force transducer (LCL454G, 0–454 g range; Omega, Stamford, CT, USA). The resistance of the ankle to dorsiflexion was measured during stepping motor-driven ankle dorsiflexion (40°; MDrive 34 with onboard electronics; microstep resolution to 256 microsteps/full step; Intelligent Motion Systems, Marlborough, CT, USA) at three different ankle-rotational velocities (40, 60 or 80°/second). The electromyography (EMG) signal was recorded from the ipsilateral gastrocnemius muscle during the same time frame. To record EMG activity, a pair of tungsten electrodes was inserted percutaneously into the gastrocnemius muscle 1 cm apart. EMG signals were bandpass filtered (100 Hz to 10 kHz) and recorded before, during, and after ankle dorsiflexion. EMG responses were recorded with an alternating current-coupled differential amplifier (model DB4; World Precision Instruments, Sarasota, FL, USA). EMG was recorded concurrently with ankle resistance measurements, both with a sample rate of 1 kHz. Both muscle resistance and EMG data were collected directly to the computer using custom software (Spasticity version 2.01; Ellipse, Kosice, Slovak Republic). Each recorded value was the average of three repetitions. The presence of spasticity response was identified as an increased ankle resistance and concurrent increase in recorded EMG activity during computer-controlled ankle dorsiflexion. To measure the contribution of the ‘mechanical’ component in the measured resistance (that is, caused by ankle ankylosis in chronically paraplegic animals), animals were anesthetized with isoflurane at the end of each recording session and the relative contribution of the neurogenic (that is, isoflurane-sensitive) and the mechanical (that is, isoflurane non-sensitive) component identified. The magnitude of the anti-spasticity effect was then expressed as the maximum possible anti-spasticity effect measured under isoflurane anesthesia minus the value of the mechanical component.

### Sensory testing

Recovery of sensory function was assessed through quantification of supraspinal ‘above-level’ escape response (AL-ER; that is, an escape or escape-attempt with incorporation of the forelimbs) thresholds to 1) a gradually increasing force to the hind paws (using the Analgesy-Meter, no disc weights added; Cat# 37215, Ugo-Basile, Collegeville, PA, USA), and 2) AL-ER latencies to a constant heat stimulus (intensity 17, cut-off at 30 seconds) to the hind paws (using a constant infrared heat source; Cat# 37360, Ugo-Basile,). The hind paw tested was gently restrained by the investigator to prevent withdrawal. For the heat perception test the apparatus was switched on ≥15 minutes prior to testing, to allow it to warm up.

For the AL-ER tests, both hind paws were tested four times, alternately, for each test, with a testing interval of ≥1 hour. No more than four measurements per day were performed, rendering two testing days per test. Maximum cut-off values for the stimuli or latency were at approximately two times that of the response threshold of uninjured animals, to prevent tissue damage. Prior to (one week) and during the experimental period, the animals are extensively habituated to the experimenter so that the animals can be held upright (loosely) during all sensory assessments. Habituation consists of picking the animal up and holding/handling it twice daily for ≥3 minutes. Subsequently, in the absence of a stimulus, animals only rarely showed escape behavior when held for the time it would take to reach cut-off values. We measured the AL-ER thresholds/latencies before injury (baseline) and every second week after injury. The final measurement was done at eight weeks post-injury. Two or less (out of the total of eight, bilateral) measurements could manually be assigned as outliers and be excluded per time point (done while blinded for time point, animal, and treatment group). In addition, individual scores were log transformed before analysis and we calculated the Maximal Possible Effect, using these log scores, as previously suggested [40]. Hence, we used the standard formula to calculate the Maximal Possible Effect, and assuming a logarithmic relation between stimulus intensity and perceived intensity:

$$100\times \frac{\log\;\left(x_{\text{final}}\right)-\log\;\left({\bar{x}}_{\text{final}\;\text{of}\;\text{SCCI}\;\text{control}\;\text{animals}}\right)}{\log\;\left({\bar{x}}_{\mathrm{baseline}\;\text{of}\;\text{SCCI}\;\text{animals}}\right)-\log\left({\bar{x}}_{\text{final}\;\text{of}\;\text{SCCI}\;\text{control}\;\text{animals}}\right)}$$

Here, *x*_*y*_ is the average AL-ER threshold of an individual animal at time point *y* (either for a thermal or mechanical stimulus).

### Magnetic resonance imaging

Eight weeks after cell grafting, rats were deeply anesthetized with 2 mg pentobarbital and 0.25 mg phenytoin (0.5 mL of Beuthanasia-D, Intervet/Schering-Plough Animal Health Corp., Union, NJ, USA) and transcardially perfused with 200 ml of heparinized saline followed by 250 ml of 4% paraformaldehyde (PFA) in PBS. A 3 cm piece of the vertebral column (Th8-L1) was placed in a tight small latex container filled with 4% PFA to prevent the formation of air bubble/tissue interface artifacts. Samples were scanned using Magnetic Resonance Imaging (MRI). Images were acquired using a 7 Tesla Bruker (Bruker Biospin Billerica, MA, USA) horizontal bore small animal magnet and a 2.5 cm imaging volume transmit/receive coil. A 3D turboRARE sequence was used with the following imaging parameters: echo time/repetition time 45/1500 ms, flip angle 180 degrees, field of view 16 × 16 × 16 mm, matrix 256 × 256 × 70 with a resulting voxel size of 62 × 62 × 229 microns. The imaging time was 84 minutes per sample.

Volume reconstructions and calculations were done using Amira software (Visage Imaging GmbH, Berlin, Germany).

### Axon counting in plastic semi-thin sections

After MRI imaging, spinal cords were dissected from the spine and a transverse (1.5-mm-thick) spinal cord block cut from the injury epicenter and prepared for plastic embedding as previously described [41]. Briefly, dissected tissue blocks were treated with 0.1% osmium tetroxide in 0.1 M non-saline phosphate buffer (pH 7.4) for 12 hours, followed by adequate rinsing in non-saline phosphate buffer. This was followed by progressive alcohol dehydration according to standard procedures up to 100% ethanol, with the addition of further dehydration in a 1:1 solution of ethanol/propylene oxide, and lastly in 100% propylene oxide. Dehydrated blocks were then prepared for resin infiltration by incubation in a 1:1 solution of resin/propylene oxide on a rotator in a fume hood overnight. The resin solution used consisted of: Eponate 12, Araldite 502, dodecenyl succinic anhydride, and 2,4,6-tri (dimethylamino-methyl) phenol (DMP-30; Ted Pella, Inc., Redding, CA, USA), mixed in ratios of 10:10:25:1, respectively. The blocks were then transferred to 100% resin for subsequent overnight infiltration on a rotator. Finally, the tissue blocks were embedded using fresh resin in multi-chamber silicone rubber molds made from a Silastic® E RVT Silicone Rubber Kit (Dow Corning Corp., Midland Township, MI, USA). The molds with embedded sections were placed in an oven at 60°C for 1 day to facilitate resin polymerization. Semi-thin (1 μm) transverse sections were then cut using a microtome (Leica Supercut RM 2065) with a 8-mm diamond knife (Histo Diamond Knife, Cat# LM 7045, DiATOME, Hatfield, PA, USA). The sections were mounted on slides with distilled water and allowed to dry on a slide warmer. Prior to staining, the slides were incubated at 60°C in an oven for 10 to 15 minutes and then contrast-stained with 4% para-phenylene-diamine (PPD).

Mosaic images were taken of two sections per animal at 20X using a Zeiss Imager. M2 fitted with a Zeiss MRm camera (Carl Zeiss Microscopy, Thornwood, NY, USA), a BioPrecision2 stage (Cat# 96S100, Ludl Electronic Products, Hawthorne, NY, USA), and Stereo Investigator software (MBF Biosciences, Williston, VT, USA). Complete mosaic images were loaded into ImageJ 1.45s. Axonal quantification involved manual definition of pixel threshold (0 to 255, grayscale; using the Triangle method). Next, ImageJ’s Analyze Particles option was used to find particles with a size of 0.20 to 250 μm^2^ and a circularity of 0.5 to 1.0 (which corresponded to axons). All acquisition and analysis values were held consistent throughout the study. Final measurements acquired were the minimal diameter (Feret’s) of each particle (and particle counts). Particles with a minimum diameter >10 μm were excluded. Employment of this parameter allowed for further axonal analysis, in which axons were divided into empirically-derived caliber sizes of small, medium, and large axons (0.3 to 1.0 μm, 1.0 to 2.5 μm, and 2.5 to 10 μm, respectively). Data were acquired per spinal region (that is, dorsal, ventral, and lateral funiculi).

### Immunofluorescence staining

After removing the 1.5 mm block from the spinal cord at the injury epicenter, the remaining caudal and rostral parts of the spinal cord (±1 cm each) were placed in 30% sucrose for cryoprotection for a minimum of five to seven days. Transverse spinal cord sections were then prepared from the L6 segment. The segment(s) in between the L6 and the injury epicenter and the one rostral to the injury epicenter were sectioned coronally and used for identification of grafted human cells. All sections were cut on a cryostat and stored free-floating in PBS with thimerosal (0.05 wt%). Sections were stained overnight at 4°C with primary human-specific (h) or non-specific antibodies in PBS with 0.2% Triton X-100: mouse anti-nuclear mitotic apparatus (hNUMA, 1:100; Millipore, Billerica, MA, USA), mouse anti-neuron specific enolase (hNSE, 1:500; Vector Labs, Burlingame, CA, USA), mouse anti-synaptophysin (hSYN, 1:2,000; Millipore), rabbit anti-glial fibrillary acidic protein (hGFAP, 1:500; Origene, Rockville, MD, USA), mouse anti-neuronal nuclei (NeuN, 1:1,000; Millipore), chicken anti-GFP (1:1,000; Aves Labs, Tigard, OR, USA), rabbit anti-anti-glutamate decarboxylase 65 and 67 (GAD65 and 67; 1:300; Millipore), mouse anti-GFAP (Cy3-labeled; 1:500; Sigma-Aldrich; St. Louis, MO, USA), rabbit anti-Ki67 antibody (mitotic marker, 1:100; Abcam, Cambridge, MA, USA), goat anti-doublecortin (DCX, 1:1000, Millipore), goat anti-choline acetyltransferase (CHAT, 1:50, Millipore/Chemicon), and rat anti human axonal neurofilament antibody (hHO14; 1:100; gift from Dr. Virginia Lee; University of Pennsylvania, Philadelphia, PA, USA). Mouse anti-growth associated protein 43 (GAP43, 1:16,000; Millipore), rabbit anti-calcitonin gene-related peptide (CGRP, 1:1,000; Biotrend, Destin, FL, USA), and rabbit anti-ionized calcium binding adaptor molecule 1 (Iba1, 1:1,000; Wako, Richmond, VA, USA), were used on the L6 transverse sections. Following washing in PBS for three to five minutes, sections were incubated with fluorescent-conjugated secondary donkey antibodies (Alexa® Fluor 488 & 647; 1:500; Jackson Immuno Research, West Grove, PA, USA; and Alexa® Fluor 555, 1:500; Invitrogen, Carlsbad, CA, USA). Sections were then mounted on slides, dried at room temperature, and covered with Prolong anti-fade kit (Invitrogen).

**Figure 2:**
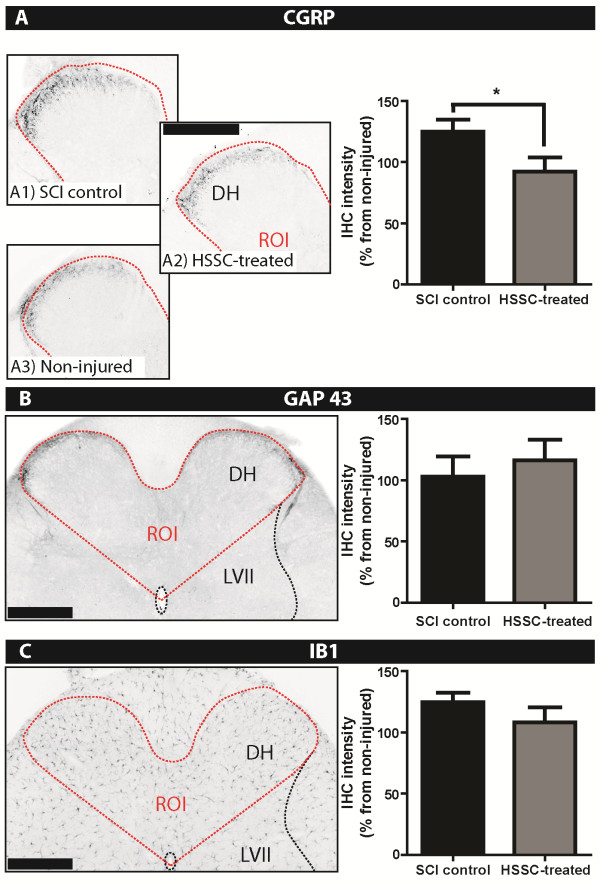
**Significant decrease in the dorsal horn CGRP immunoreactivity caudal to the injury epicenter in SCI-HSSC-treated versus SCI-control animals.** CGRP- (**A**), GAP-43- (**B**), and Iba1- (**C**) immunoreactivity in the dorsal horns (DH) caudal of the injury epicenter at two months after L3 SCI. The region of interest (ROI) was defined as outlined in B and C (left panels, red dotted line). **A:** The quantitative densitometry analysis of CGRP-immunostained images in the dorsal horns of SCI-HSSC-treated animals (A2) showed significantly decreased CGRP expression when compared to SCI-control animals (A1). **B**, **C:** The dorsal horn GAP-43 or Iba1 immunoreactivity was not significantly different between experimental groups. (**A - C:** data expressed as mean ± SEM; student *t*-tests). (Scale Bars: **A - C:** 500 μm). CRGP, calcitonin gene-related peptide; GAP-13, growth associated protein 43; HSSC, human fetal spinal cord-derived neural stem cells; Iba1, ionized calcium binding adaptor molecule 1; SCI, spinal cord injury.

Confocal images (1024 × 1024 pixels) were captured with a Fluoview FV1000 microscope (Olympus, Center Valley, PA, USA) with a 20X or 40X objective, optical section spacing of 0.5 μm, and pulse speed of 20 μsec/pixel. Other images were taken using the Zeiss Imager. M2 setup as described above, using a 10, 20 or 63X magnification. CGRP, GAP43, and Iba1 stainings on L6 transverse sections were quantified using densitometry measurements of the main dorsal horn region (Laminae I through IV; area as marked in Figure 2B). ImageJ software was used for quantification by using the Background Subtraction function.

### Statistical analyses

Behavioral data were analyzed using analysis of variance (ANOVA) one-way, or two-way group × time repeated measures, using a fixed-effect model, and a Bonferroni *post hoc* test for multiple comparisons). A *P* value of 0.05 was considered significant. Unequal variances were explored prior to using ANOVA analyses using the Bartlett’s test, but were not identified. *Post hoc* tests were only calculated if overall group differences were found. Results are expressed as means with the standard error of the mean (SEM). To analyze differences between the two groups (for example, vehicle injected versus non-injected SCI animals), we used Student’s t-tests (unequal variances were explored with the F-test, but not found) or repeated measures ANOVA. Naïve and sham operated animals were grouped (and named ‘non-injured’) in all outcomes besides the sensory tests. All statistical analyses were done using GraphPad Prism (La Jolla, CA, USA), SPSS statistics 17 (for K-Means clustering; IBM, Armonk, NY, USA), or STATA 12 (for precise post-hoc test *P*-value calculations; StataCorp LP, College Station, TX, USA) and performed two-tailed.

## Results

### General animal health and survival of animals during long-term immunosuppression

From the total of 35 SCI Sprague–Dawley rats employed in this study, 32 survived until planned sacrifice while continuously immunosuppressed; 14 NSI-566RSC-injected (1 excluded because of automutilation of hind paw on day two post-injury), 10 vehicle-injected, 8 non-injected (2 excluded, 1 because of automutilation of hind paw on day 7 post-injury and 1 because of excessive body weight loss on post-injury day 18 (likely related to immunosuppression-related toxicity)). In four surviving animals, lower extremity ulcers developed but were effectively treated with local standard triple antibiotic ointment (bacitracin, neomycin, and Polymyxin B) and cohesive bandages. In most animals, the Crede’s maneuver needed to be performed for three to five days after spinal trauma (exceptions: three animals in the NSI-566RSC-injected group and two animals in the non-injected SCI-control group, of which one died due to health issues; see above). No additional worsening (that is, a lowering in open-field locomotor scores at one day post-grafting, compared to pre-grafting values) was noted in intraspinal media- or cell-injected animals.

### Spinal injection procedure did not alter neurological outcome in previously L3-contused rats

In order to define the effect of spinal injection itself in modulating the functional recovery profile (that is, potential worsening in neurological outcome) in L3-injured animals, we first compared the effect of spinal media injection only with spinal injury animals that received no injections (10 vehicle-injected and 8 non-injected SCI animals). No significant differences were found between these two groups in any of the neurological or electrophysiological outcome measures used in this study (repeated measures ANOVA for open field locomotor scores; Student’s *t*-test for others). Based on these data, which showed no significant differences between both control groups, these two groups were then pooled into one control group and used for subsequent comparison with HSSC-grafted animals.

### Assessment of motor function

#### Gait analysis showed significant improvement in hind paw placement in SCI-HSSC-grafted animals

Gait analysis was conducted at eight weeks after grafting (or corresponding time point in controls) using the CatWalk apparatus [42]. The following parameters were analyzed: I) runway crossing time, II) rostro-caudal hindpaw positioning, III) hind paws base of support, IV) regularity index/coordination, V) stride length, and VI) phase dispersions.

#### Rostro-caudal hindpaw positioning (RCHPP)

**Figure 3:**
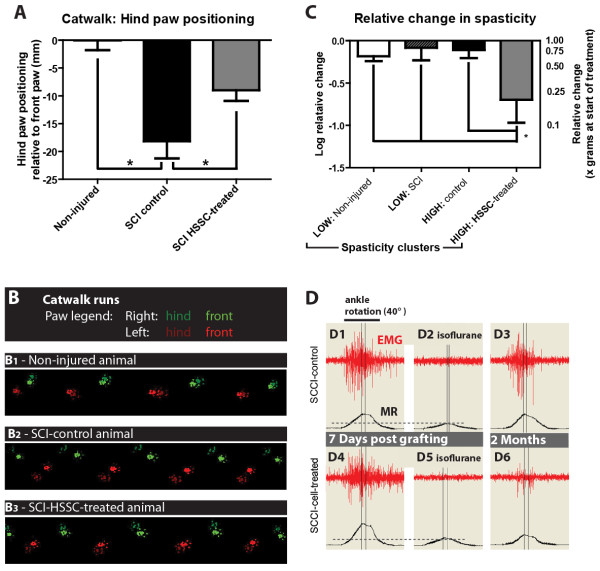
**Improvement in hind paw positioning and muscle spasticity in SCI animals grafted with HSSC. A:** CatWalk gait analysis of hind paw positioning at two months after treatment. In comparison to SCI control animals, a significant improvement was seen in HSSC-grafted animals. **B1-B3:** An example of paw step images taken from the CatWalk software in naïve (B1), SCI-control (B2) and SCI-HSSC-treated animals (B3). Note a large paw footprint overlap between the front and hind paws in naïve animals (B1) but a substantial dissociation in footprint overlap in SCI controls (B2). An improvement in paw placement in SCI-HSSC-treated animals can be seen (B3). **C:** Statistical analysis showed significant suppression of spasticity response (expressed as a muscle resistance ratio: values at two months versus seven days post injury in ‘HIGH spasticity’ HSSC-treated animals if compared to ‘HIGH spasticity’ controls). **D:** To identify the presence of muscle spasticity in fully awake animals, the hind-paw ankle is rotated 40° at a velocity of 80°/second. Spasticity is identified by exacerbated EMG activity measured in the gastrocnemius muscle and corresponding increase in muscle resistance. In control SCI animals with developed spasticity (that is, ‘high spasticity’/HIGH group), no change in spasticity response if compared to seven days post-vehicle injection was seen at two months (compare D1 to D3). In contrast to SCI control animals, a decrease in spasticity response was seen in SCI-HSSC-treated animals at two months after cell injections (compare D4 to D6). To identify mechanical resistance, animals are anesthetized with isoflurane at the end of the recording session and the contribution of mechanical resistance (which is, isoflurane non-sensitive) is calculated. (D2, D5: data expressed as mean ± SEM; one-way ANOVAs). ANOVA, analysis of variance; EMG, electromyography; HSSC, human fetal spinal cord-derived neural stem cells; SCI, spinal cord injury; SEM, standard error of the mean.

In control non-injured animals, the RCHPP was 0 ± 1.7 mm (that is, the animals are able to achieve a near complete overlap in the hindpaw positioning relative to the last ipsilateral frontpaw print; full rostro-caudal overlap is represented by a value of ‘0’). Rats receiving spinal HSSC grafts showed significantly better RCHPP, when compared to control SCI animals (−9.0 ± 1.9 versus -18.2 ± 3.1 mm, respectively, Figure 3A; Bonferroni: *P* = 0.04). Examples of the paw positioning are shown in Figure 3B for a non-injured control, SCI control, and a HSSC-treated SCI animal (Figure 3-B1, -B2, and -B3, respectively).

No significant differences were detected in other CatWalk parameters (runway crossing time, hind paws base of support, regularity index/coordination, stride length, phase dispersions), myogenic MEPs, or behavioral motor tests (open field locomotor score (modified BBB score, and regular BBB scores), single-frame motion analysis or ladder climbing test) (see Additional file 1: Figure S1A-D).

### Effective suppression of muscle spasticity in HSSC-grafted SCI animals

To identify the presence of spasticity (that is, potentiation in muscle stretch-evoked EMG activity) in animals after SCI, a computer-controlled ankle-rotational force was applied on the right or left paw in fully awake restrained animals and the resulting change in EMG activity in the gastrocnemius muscle and correlative ankle resistance was measured [39].

Independent of SCI group (control or HSSC-injected), two quantitatively different EMG patterns and corresponding resistance response (EMG/RES) patterns were recorded in spinally-injured animals. First, if compared to control non-injured animals, little or no change in EMG/RES response was seen at 1.5 weeks after SCI. Second, SCI induced an increased spasticity response in a portion of the animals at 1.5 weeks after injury. A K-Means clustering method was used to group all 44 (SCI and non-injured) animals into two groups based on the magnitude of resistance to ankle rotation at 1.5 weeks post-injury (or equivalent time point in non-injured animals). Seven animals of each SCI group (that is, control or HSSC-injected) were found to be clustered in the high ‘spasticity’ group (HIGH), which had a 31.7 ± 3.9 g increase in measured muscle resistance during ankle rotation, compared to the low ‘spasticity’ group (LOW) showing 8.9 ± 1.5 g resistance (Student’s *t*-test: *P* <0.0001). No difference in the incidence of this high ‘spasticity’ response was noted between SCI control versus cell-treated groups (incidence: *X*^2^: *P* = 0.53; extend: Student’s *t*-test: *P* = 0.24). No naïve or sham operated animals were found to be clustered in the HIGH group. Resistance to ankle rotation measured eight weeks after treatment (and expressed as relative change from 1.5 weeks post-injury values) showed a significant decrease in the HSSC-injected HIGH resistance group when compared to HIGH resistance animals from the control SCI group (Figure 3C; that is, decline of 24.8 ± 6.4 g in HSSC-injected animals and 4.8 ± 6.3 in control SCI animals; Bonferroni: *P* = 0.048).

Figure 3D shows an example of raw data depicting a post injury EMG response (red channel) and corresponding increase in muscle resistance (black channel) during ankle rotation in a SCI-control (Figure 3-D1-3) and a HSSC-injected animal (D4-6) at seven days after treatment and at the end of the eight-week survival. Clear suppression of potentiated EMG response and muscle resistance can be seen in HSSC-treated animals (compare D4 to D6). To identify and dissociate neurogenic (that is, isoflurane-sensitive) versus mechanical (that is, isoflurane non-sensitive) components, muscle resistance was re-measured after isoflurane anesthesia and the relative contribution of the mechanical component calculated. The induction of isoflurane anesthesia almost completely blocked the ankle rotation-evoked EMG response and resulting increase in muscle resistance (D2, D5).

### Assessment of sensory functions

Analysis of mechanical and thermal sensory function was performed by comparing hindpaw thresholds improvements of evoked above-level/supra-spinal withdrawal responses (that is, an escape response in which the frontlimbs and/or vocalizations are used) between experimental groups over several time points. Groups consisted of naïve control, sham operated control, SCI-control, or SCI-HSSC-injected animals. Response thresholds were measured before injury and every second week thereafter. No differences were measured between naïve and sham-operated animals at any time point in response thresholds to both mechanical and thermal stimuli (repeated measures ANOVA).

### HSSC treatment led to a significant improvement in the supraspinal perception to mechanical stimuli evoked below the level of injury

**Figure 4:**
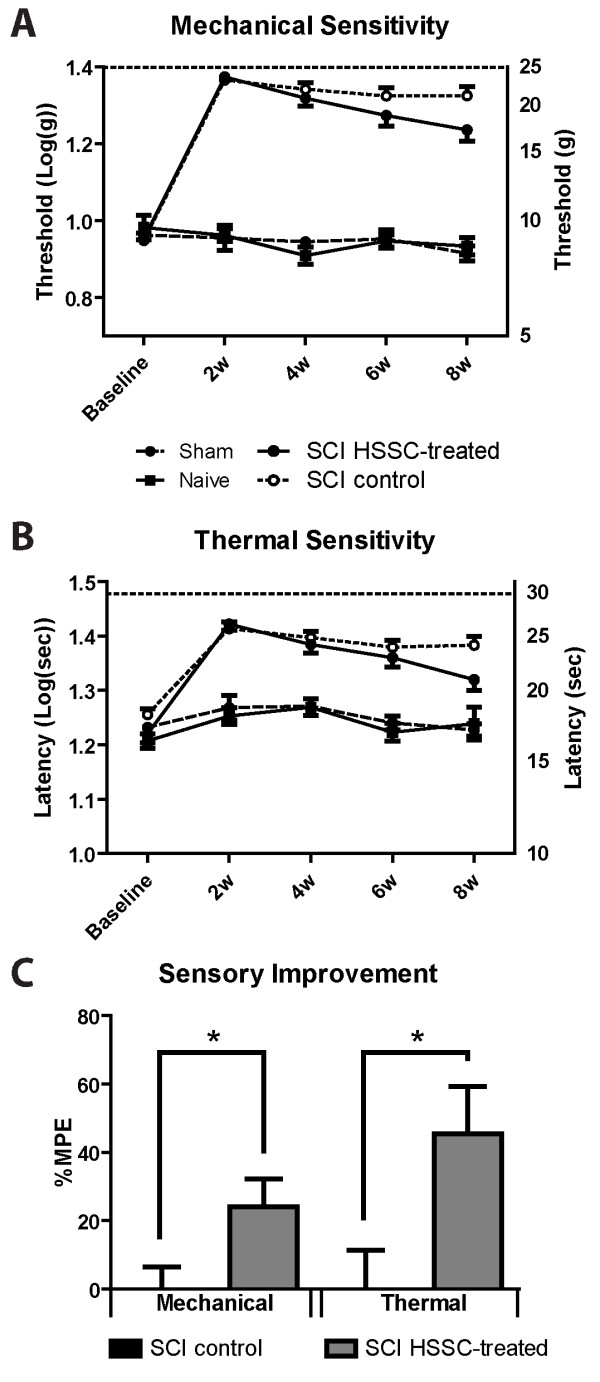
**Amelioration of hypoesthesia in SCI-HSSC-grafted animals.** Baseline and biweekly assessments of perceptive thresholds for (**A**) mechanical and (**B**) thermal stimuli, applied below the level of injury, showed a trend towards progressive recovery in SCI-HSSC-grafted animals. **C:** When expressed as percentages of the maximal possible effect for mechanical and thermal perceptive thresholds improvements, SCI-HSSC-treated animals showed significant improvements in sensory function for both mechanical and thermal components. (**A-C:** data expressed as mean ± SEM; **A-B**: repeated measures ANOVAs; **C:** Student *t*-tests). ANOVA, analysis of variance; HSSC, human fetal spinal cord-derived neural stem cells; SCI, spinal cord injury; SEM, standard error of the mean.

Prior to injury, no differences in mechanical thresholds to trigger escape responses were measured between all four experimental groups (on average 92 ± 2 g). After SCI, the thresholds increased significantly in both SCI-control and SCI-HSSC-injected animals compared to control non-injured groups, at all time points (Bonferroni; *P* <0.001). From four weeks post injury, SCI-HSSC-injected animals displayed a trend towards progressive improvement in response thresholds if compared to SCI controls (at eight weeks: 177 ± 10 g and 216 ± 10 g, respectively; Figure 4A; repeated measures ANOVA: *P* = 0.14). This resulted in a significantly higher percentage of the maximal possible effect for improvement of mechanical stimulus perception in SCI-HSSC-injected animals compared to SCI-control animals (Figure 4C; Student’s *t*-test: *P* = 0.03).

### Treatment with HSSC was associated with significant recovery of supraspinal heat perception evoked below the level of injury

Prior to SCI, measurement of thermal (infrared) stimulus-evoked paw withdrawal threshold showed no significant differences among all experimental groups (17.3 ± 0.3 seconds; one-way ANOVA). At two weeks post SCI, significant increases in paw withdrawal latencies in both the control SCI group and in SCI animals receiving spinal HSSC grafts were measured, when compared to control non-injured (sham operated and naive) groups (26.1 ± 0.7 seconds in SCI-control animals and 26.5 ± 0.7 seconds in HSSC-grafted animals versus 18.3 ± 0.2 seconds in control non-injured animals; Figure 4B; Bonferroni: *P* < 0.001).

From four weeks after treatment a trend towards a progressive normalization in response threshold was seen in HSSC-treated animals if compared to SCI controls (at eight weeks: 24.0 ± 0.9 seconds in SCI-control and 21.4 ± 0.9 seconds in HSSC-injected animals, respectively; repeated measures ANOVA: *P* = 0.09). This resulted in a significantly higher percentage of the maximal possible effect for the improvement of thermal stimuli in SCI-HSSC-injected animals compared to SCI-control animals (Figure 4C; Student’s *t*-test: *P* = 0.02).

### Postmortem spinal cord MRI showed a cavity-filling effect by grafted cells in HSSC-injected animals

**Figure 5:**
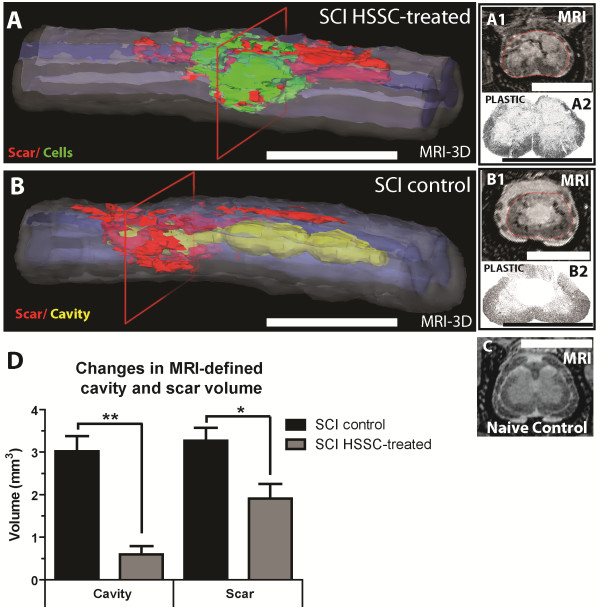
**Effective cavity-filling effect by transplanted cells in SCI HSSC-injected animals.** At the end of the two-month post-treatment survival, animals were perfusion fixed with 4% PFA, the spinal column dissected and MRI-imaged *in situ* before spinal cord dissection for further histological processing. **A, B:** Three-dimensional MRI images of spinal cord segments in animals with previous traumatic injury and treated with spinal HSSC (**A**) or media (**B**) injections. Note the near complete injected-cells cavity-filling effect in HSSC-treated animals. A1, A2, B1, B2: To validate the presence of grafted cells or cavitation at the epicenter of injury, the same region was histologically processed, semi-thin plastic sections prepared and compared to the corresponding MRI image (compare A1 to A2 and B1 to B2). **C:** Two-dimensional MRI image taken from a naïve-non-injured animal. **D:** Quantification of the cavity and scar volume from serial MRI images showed significantly decreased cavity and scar volumes in SCI-HSSC-injected animals if compared to media-injected SCI controls. (**D**: data expressed as mean ± SEM; Student *t-*tests), (Scale Bars: **A, B:** 5 mm; A1, A2, B1, B2, **C:** 3 mm). HSSC, human fetal spinal cord-derived neural stem cells; MRI, magnetic resonance imaging; PFA, paraformaldehyde; SCI, spinal cord injury; SEM, standard error of the mean.

For lesion volume analyses, a 3-cm long portion of the fixed spinal column was dissected out, kept in 4% PFA and imaged using a 7 Tesla MRI magnet. The primary goal of this analysis was to generate quantitative data on the cavity-filling effect by grafted cells and to assess the extent of rostro-caudal cavitation in vehicle-injected versus HSSC-injected animals using quantitative volume analysis (Figure 5). In vehicle-injected animals, the presence of fluid-filled cavities was readily identified as the presence of homogenous white areas and scarring as black areas (Figure 5B1; compare with non-injured: Figure 5C). In contrast, in animals receiving cell injections, the cavity was partially or completely filled with grafted cells as evidenced by the presence of low density tissue masses (Figure 5A1). The identity/presence of grafted cells in the ‘low density tissue masses’ was further validated by analysis of semi-thin plastic sections taken from the same region (compare Figure 5A2 which depicts the presence of cell grafts versus extensive cavity in Figure 4B2). Figure 5A shows a three-dimensional reconstruction image of a cell-injected animal (areas identified as grafted cells are labeled green). Figure 5B shows a SCI-control (media-injected) animal with cavity labeled in light-green-yellow.

Quantification of the cavity volume showed a significantly larger injury-induced cavity in SCI-control animals than in HSSC-injected animals (3 ± 0.4 mm^3^ versus 0.6 ± 0.2 mm^3^, respectively; Figure 5D; Student’s *t*-test: *P* <0.0001). Similarly, the scar volume seen in SCI-control animals was larger than in cell-injected animals (3.3 ± 0.3 mm^3^ versus 1.9 ± 0.3 mm^3^, respectively; Student’s *t*-test: *P* <0.001).

To assess the potential excessive grafted cell proliferation and resulting spinal cord tissue expansion, we next compared the total volume (that is, the volume of the remaining spinal cord, scar, cavity, and/or grafted cells) of the cell-grafted spinal cord segments with the corresponding segments of the control animals. The measured volumes were: 71.8 ± 3.2 mm^3^ in non-injured control animals, 54.6 ± 2.8 mm^3^ in SCI-control animals, and 59.0 ± 2.2 mm^3^ in SCI-HSSC-injected animals (Student’s *t*-test: *P* = 0.27; SCI-control versus SCI-HSSC-injected animals).

### Survival, maturation and integration of grafted HSSC

To identify the presence of human cells in the rodent spinal cord tissue, two different immunostaining/analytical methods were used. First, eGFP-tagged grafted cells were identified by the presence of GFP autofluorescence/immunoreactivity and then co-stained with neuronal and non-neuronal markers. Second, a set of human-specific antibodies was first used to validate the presence of human cells and then combined with other human-non-specific neuronal or non-neuronal antibodies.

**Figure 6:**
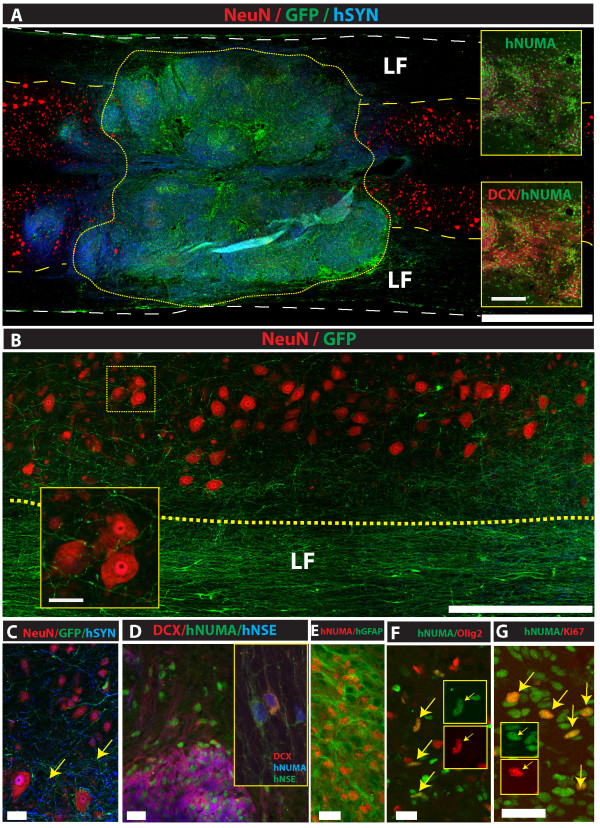
**Survival, differentiation and extensive axonal outgrowth from spinally grafted HSSC. A:** Grafted GFP+ or hNUMA+ cells can be seen almost completely filling the lesion cavity at eight weeks after grafting (yellow dotted area; inserts). **B:** Detail from ‘**A**’ depicting a dense GFP+ neurite network in the lateral funiculus (LF) and with numerous axons projecting towards α-motoneurons and interneurons in the gray matter (insert). **C:** In areas with a dense GFP+ axodendritic network, clear hSYN immunoreactivity associated with GFP+ processes can be detected (yellow arrows). **D:** The majority of grafted hNUMA+ cells showed development of the neuronal hNSE/DCX+ phenotype. **E, F:** A subpopulation of grafted hNUMA+ cells showed the astrocyte (hGFAP+) and oligodendrocyte (Olig 2) phenotype (**F;** yellow arrows). **G:** Using mitotic marker Ki67, regularly distributed hNUMA/Ki67+ grafted cells were identified (yellow arrows). (Scale Bars: **A:** 1.5 mm (inserts: 200 μm); **B:** 600 μm (insert: 75 μm); **C:** 60 μm; **D:** 20 μm; **E-G:** 10 μm). HSSC, human fetal spinal cord-derived neural stem cells; SCI, spinal cord injury.

Staining with anti-GFP, -NeuN (neuronal marker) and -human-specific synaptophysin antibody showed a near complete repopulation of the compression-induced lesion cavity by grafted GFP + cells (Figure 6A-yellow dotted area). A comparable spinal injury-cavity filling by grafted cells was seen after grafting with eGFP or non-labeled HSSC as evidenced by the presence of dense hNUMA-immunoreactive grafts (Figure 6A inserts). Analysis of axo-dendritic sprouting from grafted GFP+ cells showed that extensive rostro-caudal neurite sprouting was particularly well-developed in the lateral white matter (Figure 6B). In addition, numerous GFP+ axons branching from innervated lateral funiculi and projecting towards α-motoneurons and interneurons were identified (Figure 6B; insert). Triple staining with NeuN, hSYN and GFP antibody showed a high density of hSYN punctata in GFP+ innervated regions (Figure 6C - yellow arrows) as well as in the vicinity of endogenous NeuN+ neurons. Staining with hNUMA, hNSE and DCX antibody revealed that the majority of hNUMA+ grafted cells were DCX or DCX/hNSE immunoreactive (Figure 6A - insert; Figure 6D). Probing for glial phenotype in grafted cells by double staining with hNUMA and hGFAP or hNUMA and Olig2 antibody revealed well-developed groups of hGFAP+ astrocytes. These GFAP+ cell populations were primarily found in the white matter or at the periphery of individual DCX/hNSE+ grafts (Figure 6E). Less than 2% of hNUMA+ cells showed Olig2 immunoreactivity (Figure 6F; yellow arrows). To assess the presence of mitotically active grafted cells, sections were double-stained with hNUMA and Ki67 antibody. An estimated 0.5% to 1% of hNUMA+ cells were Ki67 positive. These double hNUMA/Ki67+ cells were regularly distributed throughout the grafted regions but no cluster(s)-like formations of hNUMA/Ki67+ cells were seen in any animal (Figure 6G; yellow arrows).

**Figure 7:**
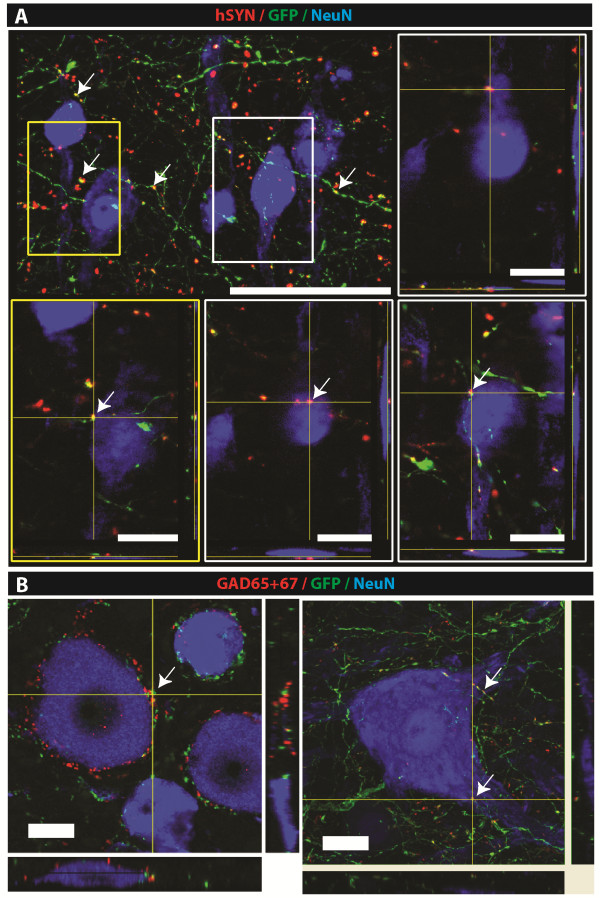
**Development of putative GABA-ergic synaptic contact between HSSC and the host neurons. A:** Confocal analysis of hSYN/GFP/NeuN-stained sections shows numerous hSYN punctata associated with GFP+ processes derived from grafted cells. Some of the hSYN/GFP+ terminals were found to be in the vicinity of the host interneurons or α-motoneurons (**A;** inserts; white arrows). **B:** Triple staining with GAD65/67/GFP/NeuN antibody showed numerous double-stained GAD65/67/GFP+ terminals residing on or in the close vicinity of lumbar α-motoneurons (white arrows). (Scale Bars: **A:** 150 μm (inserts: 30 μm); **B:** 20 μm). HSSC, human fetal spinal cord-derived neural stem cells.

Confocal analysis of spinal cord sections triple-stained with hSYN, GFP and NeuN antibodies showed numerous hSYN punctata colocalizing with GFP+ processes. Several hSYN punctata were found to reside in the vicinity of interneuronal and/or α-motoneuronal membranes (Figure 7A; inserts; white arrows). Probing for the presence of GAD65/67+ terminals derived from grafted neurons by using triple stained GAD(65/67)/GFP/NeuN sections and confocal microscopy showed the presence of GFP/GAD65/67+ terminals in the vicinity of α-motoneuronal membranes (Figure 7B; white arrows).

### Normalization of CGRP expression in L6 dorsal horns in SCI-HSSC-treated animals

To analyze changes in the spinal expression of putative central pain neuromodulators/indicators, including CGRP, GAP43 and Iba-1 [43,44], we next stained transverse L6 sections (that is, below-injury-level region) with respective antibodies at eight weeks after treatment (Figure 2A, B, C). Densitometry analysis showed that CGRP immunoreactivity was significantly reduced in SCI-HSSC-treated animals (Figure 2 A2) when compared to SCI-controls (Figure 2 A1; Student’s *t*-test: *P* = 0.04). We did not find significant group differences in IHC staining intensities of either GAP43 or Iba1 (Figure 2B, C; Student’s *t*-test: *P* = 0.58 and *P* = 0.24, respectively).

### Quantitative assessment of axonal survival in the epicenter of injury using semi-thin plastic sections

For quantitative analysis of axonal survival, a transverse spinal cord block taken from the injury epicenter was used. Using osmium/p-phenylenediamine-stained semi-thin (1-μm-thick) plastic sections, the total number of axons (divided into three subgroups based on the axonal caliber; 0.3 to 1.0, 1.0 to 2.5, and 2.5 to 10 μm in diameter) was then counted using ImageJ software.

Systematic quantification of the total number of myelinated axons counted bilaterally in a control naïve animal showed 281,352 axons (see Additional file 2: Figure S2A). Thirty-three percent was represented by small caliber axons, 57% by medium caliber axons and 10% by large caliber axons. In SCI-control animals, the total number of axons was on average 55,137 ± 5,168 and was 55,340 ± 5,650 in HSSC-injected animals (Student’s *t*-test: *P* = 0.98; Additional file 2: Figure S2B-D). Intergroup statistical analysis of the axons at specific diameters (that is, 0.3 to 1.0, 1.0 to 2.5, and 2.5 to 10 μm) showed *P*-values of 0.88, 0.84, and 0.51 (Student’s *t*-tests) between SCI-control and SCI-HSSC-grafted animals, respectively. Intergroup statistical analysis of the axons at specific funiculi (that is, dorsal, lateral, and ventral funiculi) showed *P*-values of 0.73, 0.82, and 0.72 (Student’s *t*-tests) between SCI-control and SCI-HSSC-grafted animals, respectively (see Additional file 2: Figure S2D). Additional intergroup analyses of axon survival categorized by both size and location/funiculus did not show significant differences between SCI-control and SCI-HSSC-grafted animals (*P* >0.36; Student’s *t*-tests) (see Additional file 2: Figure S2D).

## Discussion

In the present study, we investigated the treatment effect of spinally grafted GMP-grade HSSC in a L3 SCI model in rats.

HSSC were grafted into and around the epicenter of the contusion-injured L3 spinal segment at three days after spinal trauma in continuously immunosuppressed Sprague–Dawley rats. In comparison to control SCI animals with no treatment or receiving intraspinal injections of media only, the intraspinal grafting of HSSC led to a progressive and significant improvement in: I) gait/paw placement, II) muscle stretch-induced spasticity, and III) mechanical and thermal sensitivity. These behavioral benefits were associated with robust graft survival and a near complete injury-cavity-filling effect with grafted cells and corresponding lack of syringomyelia otherwise seen in control SCI cell-non-treated animals. In addition, the development of putative GABA-ergic synapses between grafted neurons and interneurons and/or α-motoneurons of the host were identified. These data demonstrate that intraspinal grafting of HSSC into an injured spinal cord segment in the acute phase of injury represents a safe and effective treatment modality. This cell-replacement therapy was effective in providing qualitatively- and quantitatively-defined functional benefits and also led to significant and long-term improvement in the structural integrity of previously trauma-injured spinal cord segments.

### Rat L3 spinal compression injury model

In our current study, a lumbar spinal injury was induced by a static 35 g pressure exerted on the dorsal surface of the L3 spinal segment by using a stainless steel-Teflon rod (2.9 mm in diameter). In our preliminary ‘survey’ study, we found that in this model the 35 g spinal cord compression needs to be maintained for a minimum of 15 minutes to produce a reproducible degree of functional and histopathologically-defined injury. These data indicate that the pathophysiological mechanism leading to neuronal/axonal degeneration in this model is primarily related to the ischemia-induced changes. However, interestingly, the histopathological changes in this model are characterized by the development of a well-delineated cavity found just below the compression site. This is in contrast to the ‘pure’ ischemia-reperfusion-induced spinal injury seen in aortic balloon occlusion models in which a selective loss of inhibitory interneurons is seen in previously ischemia-exposed spinal segments in the absence of cavity(s) formation [45,46]. In this respect, our current model appears to be similar to high velocity (weight drop model) impact injury models which show comparable cavity formation in chronic L2 or L3/4 segment-injured rats [47,48]. Similarly as demonstrated in the rat ‘weight drop’ contusion models [49], the development of spinal hyper-reflexia, as evidenced by the presence of exacerbated muscle activity evoked by computer-controlled ankle rotation, was seen in a subpopulation of injured animals in our current study. Importantly, spinal cavity formation and muscle spasticity is frequently observed in human patients with a high-velocity-impact-induced traumatic SCI [50,51].

### Rationale for early spinal cell-replacement therapy after spinal trauma

Both experimental and clinical data show that the spinal pathological processes following acute spinal injury are in part characterized by continuing axonal/neuronal degeneration, which can then continue for months to years after injury [1,52-55]. It is believed that such an ongoing axonal degeneration is, in part, the result of the lack of local trophic support associated with loss of neurons/glial cells at and around the injury epicenter. Thus, the use of treatment strategies that can replace or supplement the loss of the local neurotrophic activity and are initiated during this acute period should thus lead to a measurable treatment effect. Previous studies have demonstrated that neural stem cells of mouse, rat or human origin are a rich source of extracellularly released trophic factors (such as NGF, BDNF, GDNF, EGF, IGF-1, and VEGF) in *in vitro* cultured cells and that these cell populations retain a high level of neurotrophin expression after *in vivo* grafting in naïve animals and in a variety of neurodegenerative models including spinal injury and transgenic ALS models [56-61]. In addition, using long-term post-grafting survival periods, it was shown that following *in vivo* grafting of neural precursors with neurogenic potential into either the spinal cord at nine days post spinal cord injury, the brain at three days post ischemic insult, or the central nervous system (brain or spinal cord) of adult or developing rats, there is the development of functionally and morphologically-defined synaptic contacts between grafted neurons and the neurons of the host [62-64].

Based on these characteristics of neural precursors (NPCs), the use of NPCs for acute spinal cord grafting after trauma serves three purposes. First, it serves to provide local trophic support in the areas of previous injury (provided that grafted cells are able to home and survive long-term once grafted into the injured spinal cord milieu) and to minimize or halt the process of progressive axonal/neuronal degeneration. Second, it serves to provide a cavity-filling effect by replacing previously injured-degenerated necrotic tissue and, thus, prevents the long-term (or progressive) formation of rostro-caudal cavitations (that is, syringomyelia) [55]. Third, by the development of synaptic contact with the host axons/neurons above and below the injury level it can potentially lead to formation of a functional relay through the injury site.

### Effect of spinal grafting of HSSC on the recovery of motor function and muscle spasticity

In our current study, a combination of several motor performance tests were employed including open field modified BBB scoring, CatWalk gait analysis, inclined ladder climbing, single frame hind limb motion analysis and myogenic motor evoked potentials to identify the degree of motor function recovery after cell grafting. The changes in muscle spasticity in lower extremities (that is, below the level of injury) were also measured using a computer-controlled ankle rotational system [39]. The CatWalk gait analysis showed significantly improved paw placement in HSSC-injected SCI animals when compared to control SCI animals. In addition, a significant suppression of an otherwise exacerbated muscle spasticity response measured during ankle rotation was seen in cell-treated animals. However, no improvements in other functional CatWalk parameters (runway crossing time, hind paws base of support, regularity index/coordination, stride length, phase dispersions), MEPs, BBB score, single-frame motion analysis or ladder climbing test) were seen. Consistent with our current data, several other studies from different laboratories have demonstrated a variable degree of motor function recovery after spinal grafting of rodent or human fetal, adult or embryonic stem-cell-derived neural precursors using a variety of spinal injury models in mice and rat [14,29,32,65-72]. Importantly, these data jointly suggest that some degree of therapeutic effect can also be achieved once cells are grafted during the early post-injury period (that is, three to seven days after spinal trauma).

### Effect of spinal grafting of HSSC on the recovery of sensory function

In our study, we assessed the sensory function below the level of injury (hind paws) by measuring the mechanical and thermal thresholds for supraspinally mediated escape behavior. Using this method (in contrast to hindpaw withdrawal reflex methods) we did not observe SCI-induced hyperalgesia at the hindpaws (below-level), which is in line with observations reported from other laboratories [73,74]. We did, however, find significant improvement of both SCI-induced mechanical and thermal hypoesthesia. It is important to note that the sensory thresholds did not yet plateau at the end of the two-month survival period. We speculate that an additional quantitative and qualitative improvement in the sensory function would likely be seen should a longer post-grafting interval be studied. In addition to sensory tests, quantitative analysis of spinal parenchymal markers indicative of developing (spinal) hypersensitivity (that is, CGRP/GAP43, an indicator of aberrant sprouting of primary sensory neurons [43,70] and Iba1 staining, a marker of microglia activation [44]) were studied and showed a significant decrease in CGRP staining intensities in HSSC-treated animals if compared to SCI controls. This suggests that the recovery/decrease in sensory thresholds observed in our study is not a result of aberrant sprouting or microglia activation. Consistent with the observations from our study, previous studies from other laboratories have demonstrated similar functional and histopathologically-defined (that is, decrease in CGRP staining around the injury site) improvements after spinal grafting of fetal-tissue derived human or rodent neural or glial-restricted precursors in several mouse or rat spinal injury models [65,66,68-70,72,73].

### Differentiation of grafted cells and mechanism of HSSC-mediated therapeutic action

In our current study, a near pure population of nestin+ human fetal spinal stem cells were grafted intraspinally at three days after contusion-induced spinal cord injury. Analysis of the graft survival at two months after grafting showed a dense population of grafted hNUMA+ cells in grafted previously trauma-injured regions. In addition, numerous hNUMA+ cells which migrated out of the graft in distances ranging between 2 to 3 mm were also seen. Using human-specific antibodies against Neuron Specific Enolase and synaptophysin (markers of mature neurons), we have also shown that a majority of grafted cells developed into a neuronal phenotype. Many human specific synaptophysin+ boutons were found to reside in the vicinity of host neurons.

Quantitative analysis of the host axon survival in the injury epicenter showed no significant sparing effect in HSSC-grafted SCI animals versus medium-injected or untreated SCI animals. These data suggest that I) the majority of, if not all, axons which succumb to pathological processes resulting from secondary changes post injury, such as edema or ischemia, were already lost or irreversibly damaged at three days after trauma (that is, the time point when the cells were grafted), or II) regional cell grafting is not therapeutically effective in providing acute neuroprotection.

Analysis of the neurotransmitter phenotype in grafted cells showed the development of putative inhibitory GABA-ergic synapses with host neurons. These data show that the restoration of the local functional inhibitory circuitry by grafted cells can in part lead to the observed functional improvements. While under specific pathological conditions (such as inflammatory or neuropathic pain) spinal GABA can have excitatory effects due to reduced expression of the potassium-chloride exporter KCC2 [75,76], systematic experimental but also clinical studies have demonstrated a potent anti-spasticity effect after intrathecal treatment with the GABA_B_ receptor agonist baclofen, suggesting continuing inhibitory GABA_B_ receptor-mediated action [77,78]. In addition, we have recently demonstrated an effective anti-spastic effect after spinal parenchymal GAD65 (glutamate decarboxylase) upregulation if combined with systemic tiagabine (GABA uptake inhibitor) treatment in animals with spinal ischemia-induced muscle spasticity [79]. Jointly, these data suggest the anti-spasticity effect observed in our current study can be mediated by a synaptically coupled GABA-inhibitory effect. Accordingly, in our previous study using the same cell line, we have demonstrated the development of putative GABA-ergic synaptic contacts between grafted neurons and persisting α-motoneurons of the host in a rat spinal ischemia model. In the same animals, a significant amelioration of spasticity was measured [29]. In a recent study using electron microscopy analysis, we have confirmed the development of synaptic contacts with the host neurons at nine months after intraspinal grafting of HSSC in normal non-injured immunodeficient rats [30]. Similarly, in a more recent study, the development of functional contacts and restoration of axon potential conductivity across the region of complete Th3 spinal transection by grafted HSSC was seen [32].

In addition to restoration of the local motor circuitry, significant amelioration of otherwise increased spinal CGRP expression seen in non-treated SCI animals was measured in SCI animals receiving spinal injections of HSSC. Consistent with this observation, previous studies have demonstrated that improvement of local spinal GABA-ergic tone, as achieved by subcutaneous inoculation of a replication-incompetent herpes simplex virus (HSV) encoding GAD67 gene in a Th13 spinal cord hemisection model, led to a similar decrease in otherwise increased CGRP expression [80]. Second, previous studies have shown that spinally grafted HSSC show the expression of several trophic factors (GDNF, BDNF, and VEGF) at two months after grafting in SOD+ rats [60]. We speculate that the release of these trophic factors can potentiate the sprouting of persisting axons of the host below and above the injury and accelerate the development of new synaptic contacts particularly at longer post-grafting intervals.

Finally, we have demonstrated a near complete injury-cavity filling effect by the grafted cells at two months after grafting when the cells were grafted at three days after injury. This was in contrast to media-injected animals which showed consistent and extensive rostro-caudal spinal cord cavitation. These data suggest that early post injury cell grafting is desirable as it can effectively block the formation of the spinal cavity and its expansion and related long-term secondary spinal cord degeneration. A comparable cavity-filling effect and prevention in the progression of syringomyelia has been shown after spinal grafting of human embryonic or fetal SSCs in human patients with progressive post-traumatic syringomyelia [54,55,81].

It is important to note that the cavity-filling effect demonstrated in our current study was achieved without the use of any supporting matrices or additional topical growth factor(s) delivery. In our preliminary study, we have determined that while the density of grafted cells is relatively low to fill the cavity-forming region, the grafted cells continue to proliferate after grafting to the point where a cavity is near completely filled with grafted cells (unpublished data). The cell proliferation is inhibited once the cavity is filled and after that the cells differentiate normally. That the cells do not develop into pre-neoplastic or neoplastic cells has been assessed in a nine-month tumorigenicity study with nude rats whose Th9 spinal cord segment was first injured by contusion (manuscript in preparation). Similarly, using the same cell line as used in our current study, we have previously reported a comparable low level of mitotic activity in grafted cells at six weeks to nine months after grafting in naïve immunodeficient rats or immunosuppressed minipigs [82].

## Conclusions

In our current study, we demonstrate a functionally-defined treatment effect after spinal grafting of human GMP-grade fetal spinal stem cells in immunosuppressed SD rats with previous L3 contusion injury. This treatment effect was expressed as a significant improvement in motor and sensory function (gait/paw placement, stretch-induced muscle spasticity, and, mechanical and thermal sensitivity). No significant differences were detected in other CatWalk parameters, motor evoked potentials, open field locomotor (BBB) score or ladder climbing test. In addition, an effective filling of the trauma-induced spinal cavity with grafted cells was seen in HSSC-treated animals at two months after grafting. Jointly, these data demonstrate that the use of this clinical grade NSI-566RSC cell line with an already established favorable clinical safety profile represents a potential cell candidate for cell replacement therapy in patients with previous spinal traumatic injury.

## Abbreviations

(b)FGF: (basic) fibroblast growth factor; (c)GMP: (clinical) good manufacturing practice; (D)MEM: (Dulbecco’s) modified Eagle medium; (e)GFP: (enhanced) green fluorescent protein; AL-ER: above-level escape response; ALS: amytrophic lateral sclerosis; ANOVA: analysis of variance; BBB score: Basso, Beattie, and Bresnahan locomotion score; BDNF: brain-derived neurotrophic factor; CGRP: calcitonin gene-related peptide; CHAT: choline acetyltransferase; DCX: double cortin; DH: dorsal horn; EGF: epidermal growth factor; EMG (/RES): electromyograpy (/resistance response); FSA: foot stepping angle; G: gauge; GABA: gamma-aminobutyric acid; GAD65 and 67: glutamate decarboxylase 65 and 67; GAP43: growth associated protein 43; GDNF: glial cell line-derived neurotrophic factor; hGFAP: human-specific glial fibrillary acidic protein; hNSE: human-specific neuron specific enolase; hNUMA: human-specific nuclear mitotic apparatus; HSSC: human fetal spinal cord-derived neural stem cells; hSYN: human-specific SYNaptophysin; i.m.: intramuscular; i.p.: intraperitoneal; Iba1: ionized calcium binding adaptor molecule 1; IGF-1: insulin-like growth factor-1; IHC: immunohistochemical; KCC2: potassium chloride cotransporter 2; L1: first lumbar vertebral segment; L3: third lumbar spinal cord segment; L6: sixth lumbar spinal cord segment; LF: lateral funiculus; MEP: motor evoked potentials; MPE: maximal possible effect; MRI: magnetic resonance imaging; NeuN: neuronal nuclei; NGF: nerve growth factor; NPC: neural precursor cell; Olig2: OLIGodendrocyte lineage transcription factor; PBS: phosphate-buffered saline; PC: personal computer; PFA: paraformaldehyde; RCHPP: rostro-caudal hindpaw positioning; RHI: rump-height-index; ROI: region of interest; s.c.: subcutaneous; SCI: spinal cord injury; SD: Sprague–Dawley; SEM: standard error of the mean; SOD1: copper zinc superoxide dismutase 1; Th8: eighth thoracic vertebral segment; USP: United States Pharmacopeial Convention; VEGF: vascular endothelial growth factor.

## Competing interests

Karl Johe, PhD is an employee of and receives salary from Neuralstem, Inc. All other authors declare that no competing interest(s) exist.

## Authors’ contributions

SvG carried out or participated in all *in vivo* work, data statistical analysis, and was involved in drafting the manuscript and the design of the study. ML carried out immunohistochemistry and participated in behavioral assays. OK participated in spasticity measurements. OP and JG carried out myogenic motor evoked potential recordings. CS participated in animal surgeries and post-operative animal care. EAJ helped draft the manuscript. MH and DG participated in immunohistochemistry and microscopy analysis. SM carried out cell preparation and viability testing before grafting. KJ developed and provided the cell line used in this study. JDC and MM conceived the study, participated in its design, and drafted the manuscript. All authors read and approved the final manuscript.

## Supplementary Material

Additional file 1: Figure S1A-D Effect of spinal HSSC grafting on locomotor function (BBB), foot stepping angle, ladder climbing test and motor evoked potentials. **A**: Weekly measurement of the BBB scores modified for the L3 injuries (left y-axis) and regular BBB scores (right y-axis) showed progressive recovery in both HSSC-grafted and control SCI animals. While there was a trend toward better motor performance in HSSC-grafted-animals, this effect was not significant for both scoring systems. **B**: Single Frame Analysis showed a tendency towards regaining normal foot stepping angles between the paw and floor (measured at stance-phase initiation; see insert/drawing in B) in SCI-HSSC-treated animals. However, the angles were not significantly improved if compared to SCI controls. **C**: Using the ladder climbing test, we found a significant decrease in the number of correct steps in SCI animals if compared to naïve controls. No significant difference was seen between SCI-control and SCI-HSSC-treated animals if analyzed at two months after treatments. **D**: Motor Evoked Potentials recorded at baseline (that is, before injury) and at eight weeks post injury showed a significant decrease only for the SCI-control animals. No significant difference between HSSC-grafted and control SCI animals was detected. Click here for file

Additional file 2: Figure S2A-D Quantitative analysis of axonal survival in the epicenter of injury showed no significant differences between SCI-control and SCI-HSSC-treated animals. **A**: Schematic diagram of the axon counting design used in our current study. Axons were counted in plastic osmium-stained sections in the dorsal, lateral and ventral funiculi using ImageJ software. An example of the detection threshold to identify individual axons in a selected field is shown in A2 and A3. **B**: Transverse plastic section depicting a bilaterally distributed graft (red dashed line) and completely filling the cavity created by previous spinal compression. Note that the fusion of the graft with the host tissue is so advanced that the border between the previous injury-evoked cavity and the graft is difficult to delineate (red asterisks). **C**: An example of transverse spinal cord section taken from an animal receiving media injection. An extensive cavity occupying near completely the region of previous gray matter can be seen. **D**: Quantification of axons in SCI-control and SCI-HSSC-treated animals showed no significant differences if analyzed in dorsal, lateral or ventral funiculi or if sub-divided into axons of different caliber (S = small = 0.3 to 1.0 μm; M = medium = 1.0 to 2.5 μm; L = large = 2.5 to 10 μm). (Scale Bars: A to C: 500 μm). Click here for file
