# Supplementary figures and images for: Amelioration of motor/sensory dysfunction and spasticity in a rat model of acute lumbar spinal cord injury by human neural stem cell transplantation
Source: Stem Cell Res Ther. 2013 May 28;4(3):57. doi: 10.1186/scrt209 (PMC3706882; doi:10.1186/scrt209)

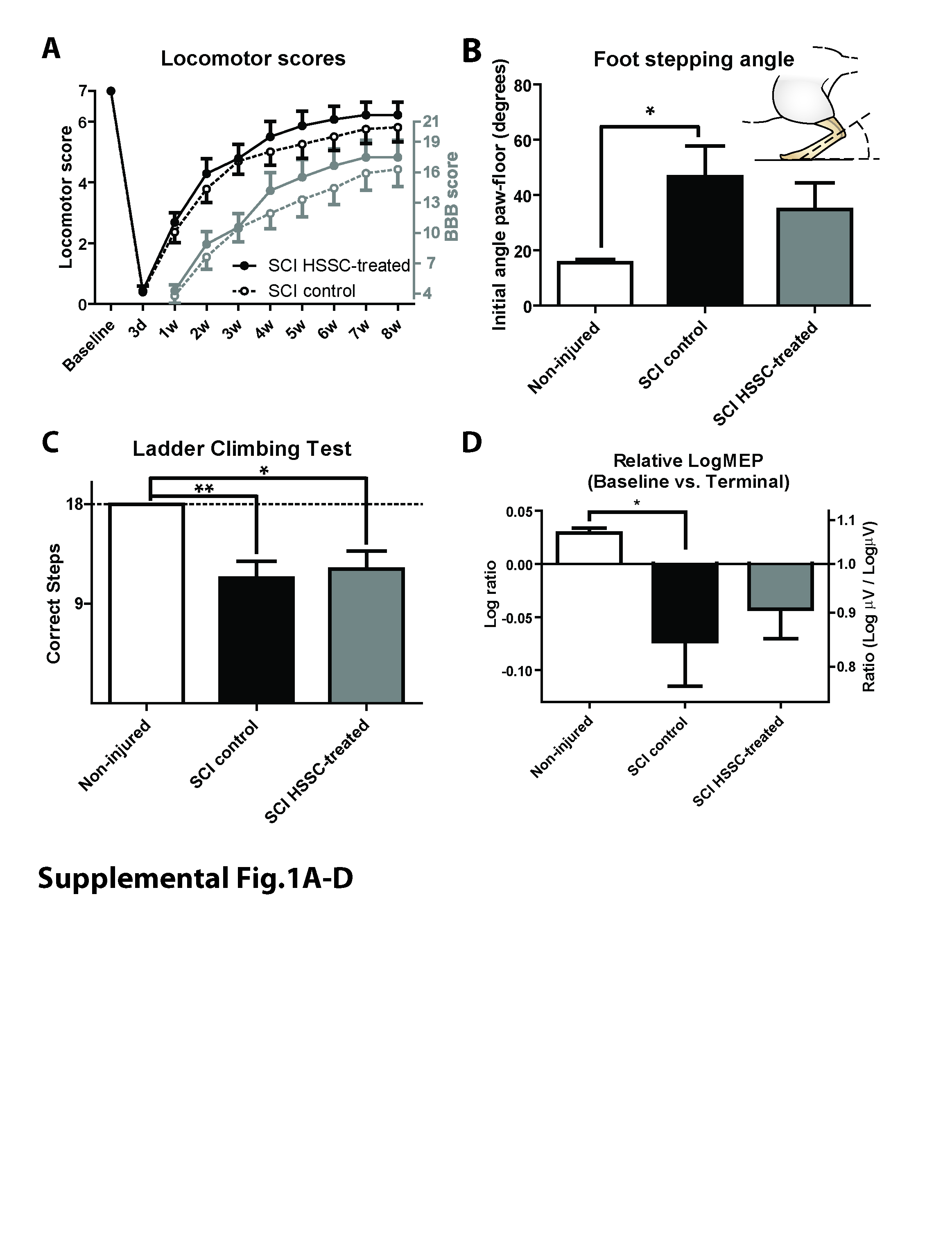

Supplement: Additional file 1: Figure S1A-D — Effect of spinal HSSC grafting on locomotor function (BBB), foot stepping angle, ladder climbing test and motor evoked potentials. A: Weekly measurement of the BBB scores modified for the L3 injuries (left y-axis) and regular BBB scores (right y-axis) showed progressive recovery in both HSSC-grafted and control SCI animals. While there was a trend toward better motor performance in HSSC-grafted-animals, this effect was not significant for both scoring systems. B: Single Frame Analysis showed a tendency towards regaining normal foot stepping angles between the paw and floor (measured at stance-phase initiation; see insert/drawing in B) in SCI-HSSC-treated animals. However, the angles were not significantly improved if compared to SCI controls. C: Using the ladder climbing test, we found a significant decrease in the number of correct steps in SCI animals if compared to naïve controls. No significant difference was seen between SCI-control and SCI-HSSC-treated animals if analyzed at two months after treatments. D: Motor Evoked Potentials recorded at baseline (that is, before injury) and at eight weeks post injury showed a significant decrease only for the SCI-control animals. No significant difference between HSSC-grafted and control SCI animals was detected. [file scrt209-S1.tiff]

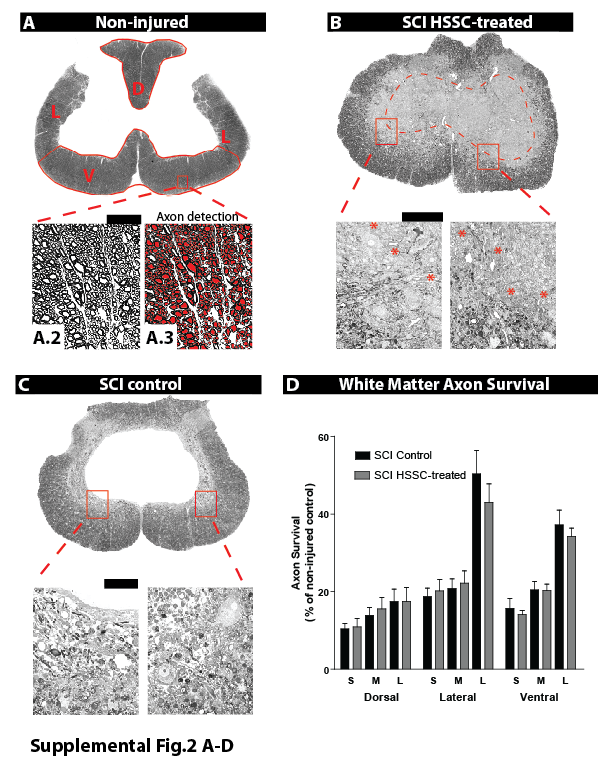

Supplement: Additional file 2: Figure S2A-D — Quantitative analysis of axonal survival in the epicenter of injury showed no significant differences between SCI-control and SCI-HSSC-treated animals. A: Schematic diagram of the axon counting design used in our current study. Axons were counted in plastic osmium-stained sections in the dorsal, lateral and ventral funiculi using ImageJ software. An example of the detection threshold to identify individual axons in a selected field is shown in A2 and A3. B: Transverse plastic section depicting a bilaterally distributed graft (red dashed line) and completely filling the cavity created by previous spinal compression. Note that the fusion of the graft with the host tissue is so advanced that the border between the previous injury-evoked cavity and the graft is difficult to delineate (red asterisks). C: An example of transverse spinal cord section taken from an animal receiving media injection. An extensive cavity occupying near completely the region of previous gray matter can be seen. D: Quantification of axons in SCI-control and SCI-HSSC-treated animals showed no significant differences if analyzed in dorsal, lateral or ventral funiculi or if sub-divided into axons of different caliber (S = small = 0.3 to 1.0 μm; M = medium = 1.0 to 2.5 μm; L = large = 2.5 to 10 μm). (Scale Bars: A to C: 500 μm). [file scrt209-S2.tiff]
